# Psychological wellbeing of children at public primary schools in Jimma town: An orphan and non-orphan comparative study

**DOI:** 10.1371/journal.pone.0195377

**Published:** 2018-04-12

**Authors:** Muluken Tigistu Hailegiorgis, Tezera Moshago Berheto, Ephrem Lejore Sibamo, Netsanet Abera Asseffa, Gashaw Tesfa, Fitsum Birhanu

**Affiliations:** 1 College of Social Sciences, Department of Psychology, Jimma University, Jimma, Ethiopia; 2 School of Public Health, College of Health Sciences and Medicine, Wolaita Sodo University, Sodo, Ethiopia; 3 College of Social Sciences, Department of Psychology, Jimma University, Jimma, Ethiopia; Center for Healthy Start Initiative, NIGERIA

## Abstract

**Introduction:**

Orphans face multiple challenges including insufficient food, shelter, schooling, and medical care. Most research on orphans in developing countries concentrates on nutrition and health status. The present study aims to explore the psychological wellbeing of in-school orphaned and non-orphaned children.

**Method:**

A comparative cross-sectional study design was used in 370 randomly selected children aged between 10 and 18. Two rosters (one for orphans and one for non-orphans) were created, and then 185 were selected from each roster. Trained field workers used structured questionnaires to obtain information from participants. An adapted Ryff Psychological Wellbeing Scale was used to measure psychological wellbeing. Mean scores were determined for each dimension and for total psychological wellbeing. The mean split was used to divide psychological wellbeing into “high” and “low”. Data were coded, entered, cleaned, and analyzed using SPSS version 20. The independent sample t-test was used to determine statistically significant differences in psychological wellbeing between orphaned and non-orphaned children. P values < 0.05 were deemed statistically significant.

**Results:**

Of 370 children, 185 (50%) were orphans. Among orphaned children, only 62 (33.5%) scored high on the total psychological wellbeing scale whereas 107 (57.8%) of their non-orphaned peers scored highly. The non-orphaned children had about 10.8 higher mean psychological wellbeing scores than their orphan counterparts (P<0.001). The mean (±SD) psychological wellbeing of the non-orphaned children was 164.0 (17.2) vs. 153.2 (17.2) in the orphaned group.

**Conclusion:**

The psychological wellbeing of orphans is significantly lower than their non-orphaned peers. Orphan support projects must consider psychosocial wellbeing in addition to material support.

## Introduction

Orphanhood leads to various problems often to prejudice, reduced access to health and school services, challenges to the basic physiologic needs and other factors that can impact their future [1]. The death of one or both parents has a profound and lifelong impact on the psychological wellbeing of children that affects every aspect of their lives; to learn, to be healthy, to play, to be productive, and to relate well to other people as they develop [2]. It is estimated that 140 million children are orphaned worldwide, 17 million due to AIDS and 90% in sub-Saharan Africa [3]. According to the 2011 Ethiopian Demographic Health Survey, 11% of children live outside the family setting with neither of their biological parents [4]. In Jimma town alone, there were over 8,000 orphans and vulnerable children in 2013 [5].

A study done in china on the psychological wellbeing of orphaned children using a sample of 1625 children aged 6 to 18 years revealed that orphaned and vulnerable children showed worse psychological wellbeing than comparison groups [6]. Research conducted on the psychological wellbeing of orphaned (n = 257) and non-orphaned (n = 140) children in Guinea revealed that orphaned children had significantly lower psychological wellbeing than non-orphans [7]. Another study by Sengendo interviewed 169 orphans and a comparison group of 24 non-orphans and found that orphans had significantly higher depression scores and lower optimism about the future than non-orphans [8]. In another Chinese study, the psychological wellbeing of orphans and non-orphans were compared (self-esteem, subjective life quality, and depression), and orphans had lower self-esteem, lower life quality, and were more depressed than non-orphans [9].

In Africa, numerous studies have been conducted on orphaned children’s health and nutritional issues; however, few studies have been conducted on psychological aspects. A cross-sectional survey conducted in Uganda revealed that orphans showed higher levels of depression and lower optimism about life compared to non-orphans. In a study conducted in Zimbabwe, orphans reported higher stress and psychosocial distress and lower psychosocial wellbeing than their counterpart. In another study, orphans were observed to be more depressed, anxious, and less optimistic and displayed angry feelings and disruptive behaviors [8,10,11]. In a study from Tanzania, 41 orphans and 41 non-orphaned controls were interviewed and orphans had increased internalization problems compared with non-orphans and 34% reported that they had contemplated suicide in the past year compared to 12% of non-orphans [7].

In Ethiopia, surprisingly few studies have examined psychological wellbeing of orphans and those exist focused on primarily AIDS-related orphans. Children orphaned by AIDS were found to have psychological problems and were deemed to be a particularly vulnerable group [12–15]. Even though there are high numbers of orphans in Jimma town, we have not found studies done on psychological wellbeing of orphans such scarcity of studies makes difficult to design and implement psychological support programs. In light of this, we aimed to explore the psychological wellbeing of orphans and compare it with non-orphans to make recommendations to program planners and strengthen existing care and support systems for orphans.

## Methods

### Study design and area

This was a comparative community-based cross-sectional study conducted in Jimma town between March 25 and April 15 2014. Jimma town is in the Oromia regional state of Ethiopia, 365 km south-west of Addis Ababa, the capital city. Based on the 2007 Ethiopian housing and population census projected to 2014, the zone had an estimated population of 280,000. The town administration has seven urban and ten town *kebeles* (smallest administrative unit in Ethiopia). There are 17 public schools in the town.

### Sampling procedures

A two-stage sampling procedure was employed to draw a representative sample of all in-school orphaned and non-orphaned children. The first stage involved selection of schools; five public primary schools were selected using a lottery method from the 14 schools in the town. Three schools were excluded from the sample as there were no orphans registered in the child clubs of these schools. The second stage involved the selection of study participants within the selected schools. Two rosters (one for orphans and one for non-orphans) were created, and then 185 were selected from each roster. This helped to keep the assumption of inhomogeneity of variance.

### Data collection and measurement

Trained research assistants collected data using a pretested structured interviewer-administered questionnaire. Psychological wellbeing, the dependent variable, is defined as the combination of feeling good and functioning effectively. An adapted Ryff Psychological Wellbeing Scale (PWB) [16] with a total of 42 items was used. The scale consists of a series of statements reflecting the six areas (dimensions) of psychological wellbeing: autonomy, environmental mastery, personal growth, positive relations with others, and purpose in life and self-acceptance. Each sub-scale consisted of 7 items. Individuals indicated their response on 6-point Likert-type scales, with higher scores on each scale indicating greater wellbeing for each dimension. The number of responses made by the subject on each question depended on whether the question was positive or negative. If it was a positive question, responses were rated from 1 to 6 where a score of 6 indicated strong agreement. If it was a negative question, scoring was performed in reverse order from 6 to 1 where 6 indicated strong disagreement. For each category, a high score indicated that a respondent has a mastery of that area in his/her life. Conversely, a low score showed that the respondent struggled to feel comfortable with that particular concept. A total PWB score was computed by adding the scores from all six dimensions. Internal consistency reliability of the local language versions of the instrument was determined for the total psychological wellbeing scale using Cronbach’s alpha. The computed Cronbach’s alpha coefficients were 0.76 for autonomy, 0.69 for environmental mastery, 0.72 for positive relation with other, 0.72 for self-acceptance, 0.62 for personal growth, 0.70 for purpose in life, and 0.86 for total PWB scale. The questionnaire was prepared first in English and then translated into Afaan Oromo (local language) with the help of language experts. To check the accuracy of the translation, the Afaan Oromo version was retranslated back to English by another person who had not had any access to the original English version of the questionnaire prior to taking up this task “S1 Questionnaire”.

The independent variables included orphanhood status and socio-demographic variables including age, sex, grade level, types of orphanhood, the causes of parental death, age at parental death, and the person(s) with whom the orphan children were currently living with.

Data were entered, cleaned and analyzed using SPSS version 20 (IBM Inc., Chicago, IL) “S1 Data”. Descriptive statistics were performed to describe the general pattern of psychological wellbeing of the respondents according to sex, age, grade level, orphan types, current situation of living, causes of parental death, and age at parental death. The independent sample t-test was computed to compare the mean difference between orphans and non-orphans in their psychological wellbeing. The mean was considered as a cutoff to classify as having a high and low score in psychological wellbeing by the mean split technique. A p-value less than 0.05 was considered statistically significant.

### Ethical statement

Ethical clearance was obtained from the institutional ethical review board of Jimma University and approval was obtained from the education office of Jimma zone before the study started. Consent was obtained from the parents or legal guardian of the children.

## Results

Of the total respondents, 185 (50%) were orphans. 146 (39.5%) of participants were male, 73 (19.7%) were orphans and 73 (19.73%) were non-orphans. The majority of study participants were aged between 10 and 14 years of age (**Table 1**).

**Table 1:** Socio-demographic characteristics of study subjects in Jimma town 2014.

Variables	Orphan	Non-orphan	Total
	Frequency (%)	Frequency (%)	
**Gender**			
Male	73 (19.7)	73 (19.7)	146
Female	112 (30.3)	112 (30.3)	224
**Age group**			
10–14	136 (36.)	147 (39.7)	283
15–18	49 (13.7)	38 (10.3)	87
**Grade level**			
5–6	102 (27.3)	71 (19.2)	173
7–8	73 (22.4)	144 (30.8)	197

Among the orphans, 105 (56.8%) were paternal, 41 (22.2%) were maternal, and 39 (21.1%) were double orphans. Twenty-four (13.0%) orphans lived with their father, 81 (43.8%) lived with their mother, 29 (15.7%) lived with no relatives, 2 (1.1%) lived in an institution, and 49 (26.5%) lived with others. By comparison, all (100%) non-orphans lived with both parents. 156 (84.3%) orphans did not know the causes of parental death. 66 (35.7%) orphans were below age five and 91 (49.2%) were above age five when their parent died. 28 (15.1%) did not know their age at the time of parental death.

With regards to the total and subscales of psychological wellbeing among study participants, the lowest mean (±SD) scores were obtained for both males 22.1 (4.4) and females 22.2 (4.3) for the sub-scale of “purpose in life”. On the other hand, male orphans scored highest on the sub-scales of autonomy, environmental mastery, and positive relations with others, while females scored highest for autonomy and positive relations with others. On the total psychological wellbeing scale, the mean (±SD) scores were 153.3 (16.9) and 153.0 (17.5) for male and female orphans and 163.3 (16.4) and 164.5 (18.0) for non-orphan males and females, respectively. The sub-scales autonomy, environmental mastery, and positive relations with others showed the highest mean scores for male and female non-orphaned children (**Table 2**).

**Table 2:** Summary statistics of the total and subscales of psychological wellbeing for orphaned and non-orphaned children in Jimma town 2014.

Orphanhood status				
Orphan			Non-orphan	
Female		Male	Female	Male
Variables	M(±SD)	M(±SD)	M(±SD)	M(±SD)
Au	27.8(4.6)	28.1(4.4)	29.7 (5.1)	29.0 (4.4)
EM	26.6(5.0)	27.4(4.5)	28.6(5.3)	29.3 (4.9)
PG	24.1(4.8)	22.1 (4.7)	25.4(4.4)	23.2 (4.3)
PR	27.4(4.5)	28.3(5.2)	30.0(4.9)	29.8 (4.6)
PL	22.1(4.4)	22.2(4.3)	23.4(4.3)	23.7 (4.3)
SA	25.0(4.5)	24.9 (4.1)	27.2(4.3)	27.8 (3.8)
PWB	153.0(17.5)	153.3(16.8)	164.4(17.9)	163.3(16.4)

**Abbreviations:** AU autonomy, EM environmental mastery, PR positive relations with others, SA self-acceptance, PL purpose in life, PG personal growth, PWB psychological wellbeing scale

Seventy-six (41.1%) and 112 (60.5%) orphaned and non-orphaned children had high scores for autonomy respectively. With regard to positive relations with others, 88 (47.6%) orphans scored high while 117 (63.2%) non-orphans scored high. Concerning self-acceptance, 113 (61.1%) orphans scored low, while 109 (58.9%) non-orphans scored high. Regarding the purpose in life subscale, 91 (49.2%) orphans scored high whereas 71 (38.4%) non-orphans scored low. For the total psychological wellbeing scale, 123 (66.5%) orphans scored low, while 107 (57.8%) non-orphans scored high **(Table 3).**

**Table 3:** The status of psychological wellbeing of orphaned and non-orphaned children.

Orphanhood status				
Orphan			Non-orphan	
High		Low	High	Low
Variables	N (%)	N (%)	N (%)	N (%)
**Au**	76 (41.1)	109(58.9)	112 (60.5)	73 (39.5)
**EM**	81(43.8)	104(56.2)	117 (63.2)	68 (36.3)
**PR**	88 (47.5)	97 (52.4)	117 (63.2)	68(36.8)
**PL**	91 (49.2)	94 (50.8)	114 (61.6)	71 (38.4)
**PG**	75 (40.5)	110 (59.5)	98 (52.9)	87(47.0)
**SA**	72 (38.9)	113(61.1)	108 (58.4)	76(41.6)
**PWB**	62 (33.5)	123 (66.5)	107(57.8)	78(42.2)

N is number, % is percentage

Using the assumption of equal variance, the t-test revealed that there was a significant mean difference in the psychological wellbeing of orphaned and non-orphaned children. Non-orphaned children had about 10.8-higher mean scores than orphans (P<0.001). Non-orphans (mean = 164.0, SD = 17.2) had higher mean than orphans (mean = 153.2, SD = 17.2; P<0.0001). The magnitude of difference in the means was large (eta-squared = 0.090). Similarly, when the dimensions of psychological wellbeing were considered, non-orphaned children had about 1.38, 1.94, 1.42, 2.16, 1.41, and 2.46 higher mean scores than orphans for the autonomy, environmental mastery, personal growth, postive relations with others, purpose in life, and self-acceptance subscales, respectively (**Table 4**).

**Table 4:** Comparison of psychological wellbeing scores between orphaned and non-orphaned children, Jima 2014.

Variables	Orphanhood status		Mean		
			Difference	t-test	p-value
	Orphan	Non-orphan			
	Mean (±SD)	Mean (±SD)			
Au	27.9(4.5)	29.3(4.8)	1.3	3.0	0.002
EM	26.9(4.9)	28.8 (5.1)	1.9	3.7	0.000
PR	27.7(4.8)	29.9(4.7)	2.1	4.3	0.000
PL	22.1(4.3)	23.5(4.2)	1.4	3.1	0.002
PG	23.3(4.9)	24.7(4.4)	1.4	2.9	0.004
SA	24.9(4.3)	27.4(4.1)	2.4	5.5	0.000
PWB	153.1(17.2)	164.0(17.3)	10.8	6.0	0.000

Grade level and parental status were significantly correlated with total psychological wellbeing and some of its components. Grade level had significant positive relationship with autonomy, environmental mastery, positive relations with others self-acceptance purpose in life, personal growth and with total psychological wellbeing. Age had a negative correlation with positive relations with others and self-acceptance.

Sex had weak positive relationship with environmental mastery, purpose in life, positive relations with others, and self-acceptance and a weak negative relationship with autonomy, personal growth, and total psychological wellbeing.

All dimensions had a significant positive relationship with each other and the total psychological wellbeing (**Table 5**).

**Table 5:** Correlation between psychological well-being and demographic measures.

	Age	Sex	Status	Grade	AU	ENV	PR	SA	PL	PG	PWB
Age	1	.095	-.021	.491**	.038	.065	-.058	-.034	.029	.008	.014
Sex	.095	1	.000	-.027	-.013	.074	037	.029	.021	-.196**	-.011
Status	-.021	.000	1	.206**	.157**	.189**	.220**	.279**	.162**	.158**	.301**
Grade	.491**	-.027	.206	1	.224**	.163**	.148**	.157**	.248**	.254**	.309**
AU	.038	-.013	.224**	.157**	1	.258**	.259**	.299**	. 354**	.293**	.640**
ENV	.065	.074	.163**	.189**	.258**	1	.232**	.271**	.324**	.384**	.658**
PR	-.058	.037	220**	.148**	.259**	.232**	1	.242**	.317**	.147**	.579**
SA	-.034	.029	.279**	.157**	.299**	.271**	.242**	1	.329**	.216**	.600**
PL	.029	.021	.162**	.248**	.354**	.324**	.317**	.329**	1	.466**	.714**
PG	.008	-.196**	.158**	.254**	.293**	.384**	.147**	.216**	.466**	1	.651**
PWB	.014	-.011	.301**	.309**	.640**	.658**	.579**	.600**	.714**	.651**	1

** Correlation significant at the 0.01 level (2-tailed)

## Discussion

Comparatively few studies have been conducted on the psychological wellbeing of children in Ethiopia but not in our study area. The present study examined the differences in the psychological wellbeing of orphaned and non-orphaned school children. It revealed a significant disparity in the psychological wellbeing of orphaned and non-orphaned school children. Orphaned children were more prone to negative psychological wellbeing than their counterparts.

In this study, orphans scored low in self-acceptance whereas non-orphans scored high. This finding is consistent with a comparative study done using Rosenberg General Self-Esteem and the Attitude Scale among 225 primary school girls [17]. The reason for such consistency could be explained by the fact that both studies were conducted among similar age groups and same setting. Regarding purpose in life subscale, orphans scored high whereas non-orphans scored low. This finding is inconsistent with a study done in India among orphanages living in an institution [18]. The variation might be attributed due to difference in the study design where the Indian study used qualitative method at which the participants better explain their psychosocial condition.

In this study, grade level and parental status were significantly correlated with total psychological wellbeing and some of its components. Grade level had significant positive relationship with autonomy, environmental mastery, positive relations with others, self-acceptance, purpose in life, personal growth and with total psychological wellbeing. This might be due to the fact that those who are about to graduate from elementary school are more likely to be accustomed with school environment, challenges, and likely to build relationship with friends around.

In our study, sex had weak positive relationship with environmental mastery, purpose in life, positive relations with others, and self-acceptance. Sex had weak negative relationship with autonomy, personal growth, and total psychological wellbeing. This might be related to men describing themselves as independent, goal oriented and competitive as compared to their compared to their counterpart also gendered process has influence on psychosocial wellbeing.

The findings of the present study are consistent with other studies conducted on the psychological wellbeing of orphaned and non-orphaned children. In a study done in Zimbabwe on 1258 orphans and vulnerable children, orphans reported higher stress and psychosocial distress and lower psychosocial wellbeing [19]. A cross-sectional study in China on 755 AIDS orphans, 466 vulnerable children, and 404 comparison children revealed that vulnerable children had the lowest level of perceived social support, where perceived social support is associated with positive psychosocial outcomes and gender and age are significant covariates of perceived social support. There was no difference in family support between the three groups [20].

Gender and age were not significantly related with any of the dimensions and the total psychological wellbeing. This finding is consistent with a comparative study done among 240 orphan and non-orphans using the Ryff psychological wellbeing scale [21]. Moreover, there was no significant differences with regard to gender and age of orphaned children in a study conducted on their psychological wellbeing in China [6]. This consistency might be related to similarity in the study tool and grade level of participants.

Several studies have shown gender-specific differences in some of the PWB dimensions. For example, women (of different ages) have scored significantly higher for positive relations [16], positive relations and personal growth [7,22], and autonomy [22]. In contrast to the present findings, gender differences in psychological wellbeing and life quality of orphaned children have been reported. A study in China included 93 orphans and 93 non-orphans and standardized instruments of depression, self-esteem, and subjective life quality were employed. Boys were found to be more vulnerable than girls in terms of their psychological wellbeing and life quality [23].

With regard to the difference between paternal, maternal, and double orphans, the current study revealed no significant difference in type of orphanhood, age at parental death, and with whom they lived except for grade level. Consistent with the current study, a cross-sectional study of 459 single orphans in family-based care in China showed that there was no significant difference between paternal and maternal orphans for all measures except that paternal orphans had more trustworthy relationship with caregivers than maternal orphans [9].

## Conclusion

The findings of this study showed that orphans are at the brink of serious psychological problems. The psychological wellbeing of orphans is significantly lower than their non-orphaned peers. Therefore, we recommend that orphan support projects need to provide not only material support but psychosocial support to attain sustainable psychological wellbeing of orphans. Moreover, societal and school authorities’ awareness needs to be created on how serious it is.

It is worth to mention the limitations of this research. This study does not include out of school orphans who may be more vulnerable and no household-level characteristics are considered. To obtain more complete understanding of respondent’s psychological wellbeing survey data might be needed. However, this study tried to use standardized scale, the first to the study setting and comparison has been made. The Ryff scale has limitation in adapted form; hence to obtain more complete understanding of respondent’s psychological wellbeing survey data might be needed. Qualitative studies may be necessary to better explore their psychosocial wellbeing and factors associated with it.

## Supporting information

S1 Questionnaire (DOCX)

S1 Data (SAV)
