# Supplementary material for: Psychological wellbeing of children at public primary schools in Jimma town: An orphan and non-orphan comparative study
Source: PLoS One. 2018 Apr 12;13(4):e0195377. doi: 10.1371/journal.pone.0195377 (PMC5896960; doi:10.1371/journal.pone.0195377)
Supplement: S1 Questionnaire — (DOCX) [file pone.0195377.s001.docx]

YUNIVARSIITI JIMMAATTI

KOLLEEJI SAAYINSII HAWAASA FI SEERA

MUUMMEE SAAYIKOLOJII

**Gaafannoon kun maanguddota iddoo sooramaa keessa jiraataniif kan qophaa,e.**

Kaayyoo qoranno kanaa yaadaatti waa’ee nagaaya xinsammuu maanguddoota soorama keessa jiran oddeefanno funaanu ta’a. Yeroo gaafanno kana guutu nagaa xinsammuu keessa maal akka fakkaatu, waan isin yaaddan ofitti amanammumaan deebii keessan yoo kennittan gaheen deebiin kee milkaa,ina qorannoo kanaatiif qabu baay’ee gudaadha .

Hub: 1. Wanti ati ibsitu hundi icittiin isaa kan eegamuudha 2. Deebii sirrii ta’e akka kennitu irra deddeebi’ee si yaadachiisa, sababii deebin sirrii hin taane qo’annoo kana waan fallessuufi.

**KUTAA1^FFA^:**

**Qajeelfama;**Gaaffiwan armaan gadittif deebi sirri ta’e kenni.

| T.lakk | Gaffiwwan | Coodii deebii | deebii |
| --- | --- | --- | --- |
| 101 | Saala | 0 = dhiira |  |
|  |  | 1 = dubartii |  |
| 102 | Umurii | 1=60-69  2=70-79  3=>80 |  |
| 103 | Sabummaa | 1=oromo  2=Amahara  3=Tigre  4=kan biro |  |
| 105 | Amantaa | 1= Ortoodoksii |  |
|  |  | 2=proteestaantii |  |
|  |  | 3= musliima |  |
|  |  | 4=kan biraa |  |
| 106 | Haala barumsaa | 1= kan hin baranne |  |
|  |  | 2=idilee dura kan barate |  |
|  |  | 3= sadarkaa 1ffaa barate |  |
|  |  | 4=sadarkaa 2ffaa barate |  |
|  |  | 5= kanaa ol kan barate |  |
| 108 | Waggaa meeqaaf as jiraattan waggaa.................. | |  |

**KUTAA 2^FFA^**: **gaaffii waa’ee nagaan xinsammuu (psychological well-being)**

**Qajeelfama:** Gaaffiwwan armaan gadii tokkon tokkoon dubisii sirritti dhugaadha kan jettu murtii mataa keeti kenni.

|  |  | B.hin.galu | Hamma ta,e | Xiqqoo wali | xiqqoo | Hamma ta,e | B.waligala |
| --- | --- | --- | --- | --- | --- | --- | --- |
| 1 | Yaada koo ibsachuuf hin sodaadhu,yoo namoonni bay,eenuu nafallessaniiyuu. | 1 | 2 | 3 | 4 | 5 | 6 |
| 2 | Waliigala,ana kan natti dhaga,amu fedhi ani keessa jiraadhu. | 1 | 2 | 3 | 4 | 5 | 6 |
| 3 | Ani fedhii hojii sarara koo bal,su hin fedhu. | 1 | 2 | 3 | 4 | 5 | 6 |
| 4 | Namni baay,een ana kan na ilaalu nama jaalatamaa fi amanamaa dha. | 1 | 2 | 3 | 4 | 5 | 6 |
| 5 | Yemmuun seenaa jireenya kiyyaa, wantootni akkamitti akkan dabarse nagammachiisa. | 1 | 2 | 3 | 4 | 5 | 6 |
| 6 | Murteen kiyya yeroo hundaayyuu dhibbaan hoomaatuu narra hin gahu ,abbaan fedhe waan fedhe yoo hojjeteeyyuu | 1 | 2 | 3 | 4 | 5 | 6 |
| 7 | Fedhii jireenya kiyyaa guyyaa hundaa gadi deema. | 1 | 2 | 3 | 4 | 5 | 6 |
| 8 | Ani kanan yaadu muuxannoo haara,a qabaachuun barbaachisaadha,innis rakkoo akkamitti akka waa,ee koo fi addunyaa hiiku yaadduf. | 1 | 2 | 3 | 4 | 5 | 6 |
| 9 | Walitti dhufeenya cimaa uumuun natti cima . | 1 | 2 | 3 | 4 | 5 | 6 |
| 10 | Ani kallattii fi sababa jireenyaa qaba. | 1 | 2 | 3 | 4 | 5 | 6 |
| 11 | Waliigala ofitti amanamummaa fi ilaalcha gaariin ofii qaba. | 1 | 2 | 3 | 4 | 5 | 6 |
| 12 | Ani namni biraa maal naan jedha jedheetan yaada. | 1 | 2 | 3 | 4 | 5 | 6 |
| 13 | Ani namoota waliin jiraadhu waliin wal hin gitne akkasumas hawaasa waliin. | 1 | 2 | 3 | 4 | 5 | 6 |
| 14 | Akka waa,ee waan tokkoo yaaddu ,ani hoomayyuu hin fooyyessine akka nama waggaa bay,ee | 1 | 2 | 3 | 4 | 5 | 6 |
| 15 | Ani yeroo darbe kophumaa natti dhaga,ma hiriyaa waa,ee dhimma koo himadhu hin qabu. | 1 | 2 | 3 | 4 | 5 | 6 |
| 16 | Hojiin kiyya guyyaa guyyaan yeroo baay,ee kan gatii hin qabne barbaachisaa kan hin taanedha. | 1 | 2 | 3 | 4 | 5 | 6 |
| 17 | Ani akkuma namoota kaanii kan natti dhaga, amu, jireenya kanan qabu caalaan balleesse. | 1 | 2 | 3 | 4 | 5 | 6 |
| 18 | Namni yaada cimaa qabu dhibbaa narraan gaha. | 1 | 2 | 3 | 4 | 5 | 6 |
| 19 | Ani nama bay,ee cimaadha, jireenya koof kanan ittigafatamamuufi sireessudha. | 1 | 2 | 3 | 4 | 5 | 6 |
| 20 | Ana kan nagammachiisu namootan waliin jiraadhu waliin haasa,uudh. | 1 | 2 | 3 | 4 | 5 | 6 |
| 21 | Ani wantin jireenya koo keessatti hojjedhu,na hin gammachiisu. | 1 | 2 | 3 | 4 | 5 | 6 |
| 22 | Ani amala koo irra caalaa nan jaaladha. | 1 | 2 | 3 | 4 | 5 | 6 |
| 23 | Ani yaada kootti ofitti amanamummaan qaba. | 1 | 2 | 3 | 4 | 5 | 6 |
| 24 | Ani kanan yaadu ittigaafatamummaa akka malee natti bay,achuudha. | 1 | 2 | 3 | 4 | 5 | 6 |
| 25 | Ani itti hin gammdne haala haaraa na barbaachisu,waan tokko haala duraaniirra. | 1 | 2 | 3 | 4 | 5 | 6 |
| 26 | Namoonni kan na ibsaan nama nama gargaarufi yeroo kiyya namoota biraa waliin dabarsuuf fedhi akkan qabu. | 1 | 2 | 3 | 4 | 5 | 6 |
| 27 | Ana karoora gara fuulduraa baasuun na gammachiisa akka karoorsettis nan hojjedha. | 1 | 2 | 3 | 4 | 5 | 6 |
| 28 | Karaa baay,edhaan kan natti dhaga,amu hojiin kiyya akka najalaa kufee fi itti hin gammadne. | 1 | 2 | 3 | 4 | 5 | 6 |
| 29 | baay,ee natti ulfaata sagalee kennu yaada dhimma walmakaa irratti. | 1 | 2 | 3 | 4 | 5 | 6 |
| 30 | Ani jireenya koo sirreessuu natti ulfaata akka haala natti toluun. | 1 | 2 | 3 | 4 | 5 | 6 |
| 31 | Anaaf jireenyi adeems itti fufaa waa baruuti,jijjiiramaa fi guddinaati. | 1 | 2 | 3 | 4 | 5 | 6 |
| 32 | Ani muuxxannoo walitti dhufeenyaa oo,aa nama biraa waliin hin qabu. | 1 | 2 | 3 | 4 | 5 | 6 |
| 33 | Namni tokko tokko kaayyoo malee jiraachuu ni jajjabeessa, ani garuu miti. | 1 | 2 | 3 | 4 | 5 | 6 |
| 34 | Ani of kanan ajaju akkan yaade fi na barbaachisetti malee akka namni biraa itti yaade fi nibarbaachisa jedhetti miti. | 1 | 2 | 3 | 4 | 5 | 6 |
| 35 | Ani osoo mana ijaaradhee haala jireenya koo foyyeessuu nan jaaladha. | 1 | 2 | 3 | 4 | 5 | 6 |
| 36 | Ani fooyya,iinsa guddaa fiduuf shaakalaan jira, jijjirama jireenya koo yeroo yeroodhaan. | 1 | 2 | 3 | 4 | 5 | 6 |
| 37 | Ani beeka hiriyoota nan fedha isaanis nafedhu. | 1 | 2 | 3 | 4 | 5 | 6 |
| 38 | Yegguun ofii kiyya nama biraatiin of dorgomsiisu,hiriyaa,naman xiqqoo beeku, waa,een koo waan gaarii natti dhaga,ama. | 1 | 2 | 3 | 4 | 5 | 6 |

Jimma University

College of social science and law

Department of psychology

The purpose of this questionnaire is to gather information regarding to psychological wellbeing of orphan and non-orphan children. This questionnaire has two parts: the first part has demographic questions about the respondents; the second part has Ryff’s Scale of Psychological Wellbeing Scale.

The information you provide has a very important input in the direction and completion of this study, so please try to be honest, and careful. There is no one to judge you because there is not right or wrong answer for the questions.

The information will be kept confidential and be only applied for the study. Yours right information helps to reach the goals of the study.

Thank you for investing your time and honesty completing this questionnaire.

Part one፡ Background Information

Direction: please indicate your answer by making ( ) in the box that corresponds to your answer or to write the correct answer on blank space

1. Age ________________

2. Sex A. Male B. Female

3. Grade level ______________________________

4. Do your parents alive?

A. Yes B. no

5. Your answer for question 4 no which parent is missed

A. Father B. Mother C. Both

6. What is the cause of parental death?.................................................

7. What was your age at parental death ……………………………………………..

8. Current living

A. With father B. With mother

C. With relatives D. With non-relatives E. Institutions F. With both parents G. Other ________________

RYFF SCALES OF PSYCHOLOGICAL WELL-BEING

The following set of statements deals with how you might feel about yourself and your life. Please remember that there are neither rights nor wrong answers. Put  mark that best describes the degree to which you agree or disagree with each statement.

| put  mark that best describes the degree to  which you agree or disagree with | Strongly  Disagree | Disagree | Disagree  Slightly | Agree  Slightly | Agree | Strongly  Agree |
| --- | --- | --- | --- | --- | --- | --- |
| 1. I am not afraid to voice my opinions, even when they are in opposition to the opinions of most people. |  |  |  |  |  |  |
| 2. In general, I feel I am in charge of the situation in which I live. |  |  |  |  |  |  |
| 3. I am not interested in activities that will expand my horizons. |  |  |  |  |  |  |
| 4. Most people see me as loving and affectionate. |  |  |  |  |  |  |
| 5. When I look at the story of my life, I am pleased with how things have turned out. |  |  |  |  |  |  |
| 6. My decisions are not usually influenced by what everyone else is doing. |  |  |  |  |  |  |
| 7. The demands of everyday life often get me down. |  |  |  |  |  |  |
| 8. I think it is important to have new experiences that challenge how you think about yourself and the world. |  |  |  |  |  |  |
| 9. Maintaining close relationships has been difficult and frustrating for me. |  |  |  |  |  |  |
| 10. I have a sense of direction and purpose in life. |  |  |  |  |  |  |
| 11. In general, I feel confident and positive about myself. |  |  |  |  |  |  |
| 12. I tend to worry about what other people think of me. |  |  |  |  |  |  |
| 13. I do not fit very well with the people and the community around me. |  |  |  |  |  |  |
| 14. When I think about it, I haven't really improved much as a person over the years. |  |  |  |  |  |  |
| 15. I often feel lonely because I have few close friends with whom to share my concerns |  |  |  |  |  |  |
| 16. My daily activities often seem trivial and unimportant to me. |  |  |  |  |  |  |
| 17.I feel like many of the people I know have gotten more out of life than I have. |  |  |  |  |  |  |
| 18. I tend to be influenced by people with strong opinions. |  |  |  |  |  |  |
| 19. I am quite good at managing the many responsibilities of my daily life. |  |  |  |  |  |  |
| 20. I enjoy personal and mutual conversations with family members or friends. |  |  |  |  |  |  |
| 21. I don't have a good sense of what it is I'm trying to accomplish in life. |  |  |  |  |  |  |
| 22. I like most aspects of my personality. |  |  |  |  |  |  |
| 23. I have confidence in my opinions, even if they are contrary to the general consensus. |  |  |  |  |  |  |
| 24. I often feel overwhelmed by my responsibilities |  |  |  |  |  |  |
| 25. I do not enjoy being in new situations that require me to change my old familiar ways of doing things. |  |  |  |  |  |  |
| 26. People would describe me as a giving person, willing to share my time with others. |  |  |  |  |  |  |
| 27. I enjoy making plans for the future and working to make them a reality. |  |  |  |  |  |  |
| 28. In many ways, I feel disappointed about my achievements in life. |  |  |  |  |  |  |
| 29. It's difficult for me to voice my own opinions on controversial matters. |  |  |  |  |  |  |
| 30. I have difficulty arranging my life in a way that is satisfying to me. |  |  |  |  |  |  |
| 31. For me, life has been a continuous process of learning, changing, and growth. |  |  |  |  |  |  |
| 32. I have not experienced many warm and trusting relationships with others. |  |  |  |  |  |  |
| 33. Some people wander aimlessly through life, but I am not one of them |  |  |  |  |  |  |
| 34. I judge myself by what I think is important, not by the values of what others think is important. |  |  |  |  |  |  |
| 35. I have been able to build a home and a lifestyle for myself that is much to my liking. |  |  |  |  |  |  |
| 36. I gave up trying to make big improvements or changes in my life a long time ago. |  |  |  |  |  |  |
| 37. I know that I can trust my friends, and they know they can trust me. |  |  |  |  |  |  |
| 38. When I compare myself to friends and acquaintances, it makes me feel good about who I am. |  |  |  |  |  |  |
